# University of California Research Seminar Network: A Prospectus

**DOI:** 10.1371/journal.pbio.1000289

**Published:** 2010-01-19

**Authors:** James R. Carey, John Christian Laursen, Steven D. Glaser, Stephen Raphael, Gregory H. Miller, John Crawford, Timothy F. Lane, Patricia J. LiWang, Kimberly Hammond, Theodore Groves, Jean-Francois Pittet, David Stuart, Phokion G. Kolaitis, Laura Serwer, Mengfei Chen, Kenneth Feer

**Affiliations:** 1 Department of Entomology, University of California Davis, Davis, California, United States of America; 2 Department of Political Science, University of California Riverside, Riverside, California, United States of America; 3 Department of Civil and Environmental Engineering, University of California Berkeley, Berkeley, California, United States of America; 4 Department of Public Policy, University of California Berkeley, Berkeley, California, United States of America; 5 Department of Applied Science, University of California Davis, Davis, California, United States of America; 6 Department of Arts and Dance, University of California Irvine, Irvine, California, United States of America; 7 Department of Obstetrics and Gynecology, University of California Los Angeles, Los Angeles, California, United States of America; 8 School of Natural Sciences, University of California Merced, Merced, California, United States of America; 9 Department of Biology, University of California Riverside, Riverside, California, United States of America; 10 Department of Economics, University of California San Diego, La Jolla, California, United States of America; 11 Department of Anesthesia, University of California San Francisco, San Francisco, California, United States of America; 12 Department of Physics, University of California Santa Barbara, Santa Barbara, California; 13 Department of Computer Science, University of California Santa Cruz, Santa Cruz, California, United States of America; 14 School of Pharmacology, University of California San Francisco, San Francisco, California, United States of America; 15 School of Social Sciences, University of California Irvine, Irvine, California, United States of America; 16 College of Health Sciences, University of California Irvine, Irvine, California, United States of America; 17 University of California Office of the President, Oakland, California, United States of America

## Abstract

By webcasting the hundreds of seminars presented in the University of California system each week, UC educators hope to enhance the exchange of scientific information for their campuses and create the foundation for an international research seminar network.

The infectious enthusiasm of scholars speaking about their research is often perfectly complemented by the never-ending quest of academic audiences for new knowledge, making seminars one of the most forceful and efficient mechanisms for transmitting scholarly information. Indeed, seminar attendance is an integral part of the experience for University of California (UC) researchers, with an estimated 300 to 500 seminars during a typical week of the academic year across 900 departments or programs in the UC system. This translates to well over 10,000 seminars annually that are presented in diverse formats and various frequencies—weekly department, graduate group and center seminars, monthly or quarterly talks in distinguished scholar lecture series, and annual university lectures by eminent faculty.

Although the importance of sponsoring and attending research seminars is universally acknowledged by UC scholars and administrators, time and travel constraints limit the potential for maximizing transmission and exchange of the massive amount of information contained in these seminars. The schedules of researchers (particularly faculty) are often packed so tightly that they cannot accommodate the additional time needed to walk to many seminars on their own campus, let alone travel to a neighboring campus. Consequently, the number of UC researchers who attend seminars on UC campuses other than their own is negligible.

The UC Seminar Network was conceived as a way to address these time constraints and open up numerous new possibilities for information exchange by delivering scientific presentations to a researcher's office computer using webinar technology that links seminars across the 10-campus UC system. This network would increase intra-, inter-, and off-campus seminar access, encourage speaker sharing, reduce travel, augment outreach, and provide electronic feeds for on-demand streaming and archiving.

## Background

To provide both context and perspective for creation of the UC Seminar Network, we briefly describe four seminar webcasting enterprises. (1) webcast.berkeley (http://webcast.berkeley.edu/) is a lecture webcasting system developed by the UC Berkeley Multimedia Research Center in conjunction with UCB's Education Technology Services (ETS) to stage and videotape on-site events and publish them online for on-demand viewing. The UC Regents retain the copyright to all media recordings. (2) University of California Television (UCTV) (http://www.uctv.tv/ondemand/) is a non-commercial channel featuring programming from throughout the UC system. Programs, transmitted by television or through the Web, include documentaries, faculty lectures, research symposia, and artistic performances, as well as postings related to K–12 education and a Med Ed Hour that offers up-to-date research on health and medicine. (3) Technology, Entertainment, Design (TED)—these 4-day conferences (http://tinyurl.com/kpmvbo), run by the not-for-profit Sapling Foundation in New York City, have a strict time limit of 18 minutes, incorporate no audience interactions, include few or no PowerPoint slides, and emphasize speaker engagement at a scientifically untutored listener level. Presentations are delivered, recorded and edited using state-of-art technology, with extraordinarily high quality products posted for on-demand viewing. (4) National Institute of Health (NIH) VideoCasting—the Center for Information Technology (CIT) makes special events, seminars, and lectures available on the NIH network and their VideoCast website (http://videocast.nih.gov/PastEvents.asp). The talks by invited scientists (typically 50-minute PowerPoint presentations followed by 5–10 minutes for questions) feature original research in, for example, biology, medicine, and bioethics.

Despite the differences in speakers, topics, production quality, and search and indexing capabilities across these four seminar webcasting enterprises, the seminars suggest what will be possible with a full-scale UC seminar network. Indeed the veritable treasure-trove of seminars that would be available in a UC Seminar Network can be glimpsed from the offerings of webcast.berkeley alone, which features topics ranging from the Mars Rover mission, the great transitions in evolution, and emerging infectious diseases and global health to those involving equity and equality, war and presidential politics, and the constitution, the military, and political accountability.

The seminars available at these four websites differ from those that will be produced in the proposed UC Seminar Network in at least two respects. First, whereas most speakers featured in the two UC operations and in nearly all the TED webcasts are distinguished scholars and prominent policy makers, the speakers in the UC Seminar Network series will also include junior speakers. Second, unlike the use of state-of-the-art technology for both producing and recording the seminars in the UC operations and by TED, excepting seminars in elite series, the technology used in the UC Seminar Network will generally be off-the-shelf and thus less high-tech—a necessary concession to allow universal implementation.

## Overview

### Piloting

A seminar webcasting pilot project was conducted at UC Davis in 2009 during the spring and fall terms, the purpose of which was to field-test live streaming and recording seminars with special emphasis on ease of use of the basic equipment and software, efficiency of set up and recording mechanics, and note reactions of both the speaker and the audience. Because of the emphasis on testing rather than promotion, the webcasting aspect of the seminars was only minimally advertised. A total of 49 seminars or presentations were webcast including 16 in the Department of Entomology, eight in the Graduate Group in Ecology and Evolution, and 25 from a two-day conference hosted by the Humanities Digital Institute.

The piloting results laid to rest a number of concerns about seminar webcasting that were raised a priori. For example, there was no fall-off in attendance due to the accessibility of seminars via live-streaming or on-demand, only two of 49 speakers did not “opt in” to the webcasting, and the audiences were universally supportive of the webcasting operation with no evidence of distractions due to the presence of the technology and videographer. The pilot project shed light on a number of operational aspects of webcasting including the importance of compact and thus portable equipment, of adopting basic “best practices” protocols including speaker requirements for microphone use and movement restrictions (i.e., stay near podium), of basic checklists to reduce the risk of mistakes, and use of background drapes and/or podium logos to identify the department/university as well as to provide a clean and professional looking set. An important operational principle applied to all seminars was that any problems that were encountered with the equipment were not allowed to delay seminar start-times.

### Beneficial Byproducts

It's likely that a UC seminar network would improve the quality of many seminar, as speakers often take talks more seriously when they know their presentation will be recorded and transmitted widely. Additionally, invited speakers and job applicants could be prescreened from their video-streamed or on-demand seminars. On-demand videos could also be used in training modules for undergraduate, graduate, and professional students. Finally, a seminar network would heighten UC scientist's awareness of the remarkable diversity of off-campus UC research sites with talks originating from locations ranging from the Scripps Institute of Oceanography in La Jolla and the Los Alamos National Laboratory in New Mexico to the Burns Piñon Ridge Reserve in the Mohave Desert and the Gump South Pacific Research Station in French Polynesia.

### Open Access

Inasmuch as most UC research is funded by taxpayers, it can be argued that the public should have direct if not immediate access to the results [1]. This is currently the case with some of the open-access scientific journals such as the Public Library of Science (PLoS) and should be considered when developing the proposed system to link seminars across the UC System. For example, people might wish to listen to a seminar speaker who presents new results on a family illness or on wellness concepts. Or, people who have serious hobbies, such as amateur astronomers, reptile hobbyists, or bird-watching enthusiasts, may wish to view a scientific seminar. People who do not read scholarly articles can also benefit indirectly from open-access seminars. For example, patients benefit when their doctor has access to the latest research, growers benefit when their farm advisors have access to experts speaking about the most effective pest control technologies, and the public benefits when legislators and their staff have direct access to cutting-edge scientific information. Science teachers who work at schools with high proportions of minorities could be alerted to seminars to be presented by minority scientists and thus expose his/her students to potential role models via webcast seminars. Seminars of general interest could also be publicized in local newspapers, along with a website address listing scheduled seminar details and URL sites.

### Future Prospects

As other university systems and institutional clusters adopt a similar idea for making their seminars available via webinar technology, the *UC Seminar Network* would become part of a *University Seminar Network* which, in turn, could become part of a global *Research Seminar Network*. Because many thousands of weekly seminars across all time zones and areas of science would be listed on an international website, the postings would need to be sorted by topic or key word and/or be listed according to interest group (e.g., malaria), time zone/region, and language. As researchers become more comfortable with the webinar concept, seminar series can begin being organized in new configurations involving institutions in different time zones and on different continents (e.g., Continental/Hemispheric Seminar Series) with a mid-morning seminar presented in Berkeley being viewed as an early evening seminar in Tel Aviv.

Research seminars are concerned with transmitting new knowledge rather than, for example, disseminating marketing data as in business seminars or delivering information on nutrition in public health seminars. Given the central role of research at UC, expanding the scale of research seminar webcasting and developing a more comprehensive infrastructure for their access, cataloging, and storage has the potential to increase both the number and specificity (relative to interests and expertise) of seminars available to UC researchers and thus enhance the flow and exchange of new knowledge within the system. Of course, the concept of using webinar technology is not new to the UC system since, as noted previously, the not-for-profit TV channel UCTV as well as the webcast.berkeley program, have been recording and transmitting high-end seminars for a number of years. By providing live video streams and on-demand records of all seminar types, the UC Seminar Network would complement both of these webcasting enterprises, with each representing a component of a larger communications enterprise.

As part of one of the world's largest and most prestigious library systems, the California Digital Library (CDL) would clearly play a central role in developing the seminar network by providing expertise on how best to catalog, store, preserve, and retrieve recorded seminars. It is conceivable that video records of scientific seminars may become a bibliographic (and thus both searchable and citeable) concept much like that for many news organizations, complete with on-demand videos showcased as *Editor's Picks*, *Most Viewed*, and *Highlights of Week*. Indeed, the recorded seminars can become part of the liquid fabric of all information contained within a universal library infrastructure, fostering a new culture of interaction and participation.

As the research arm of the state, the University of California is currently exploring the concept described here concerned with linking seminars systemwide as a mechanism to both enhance and accelerate research. Although current budgetary issues as well as technological constraints may limit development in the short term, continued field testing of seminar webcasting can still vet the concept, quantify the level of interest, and evaluate the technology. In light of trends in science, technology, the Internet in general, and the on-going seminar webcasting of a number of programs including NIH, TED, UCTV, and webcast.berkeley, it is likely that the seminar network system described here or a close variation thereof will begin to emerge throughout the research communities and eventually merge into national and then global networks of research seminar interconnectivity.

**Box 1. How Webcasting Works**

*Webcasting* is a generic concept referring to the transmission of linear audio or video content over the Internet and can occur in a number of forms [2]. These include a *web conference* consisting of many-to-many connections, a *webinar* consisting of one-to-many connections (i.e., speaker to audience), and *podcasts* (usually) referring to episodic releases of webcast lectures or webinars. A webcast is created through the use of special *web conferencing software* such as the multimedia program Adobe Acrobat Connect Pro, which electronically streams encoded video/audio data from a lecture to a server and then to an online audience. The different types of webcasts are distributed from the *web server* as either a file or a stream, the first providing the user the ability to play the webcasts multiple times offline (e.g., media players) and the latter allowing the user to skip portions of the file without completely downloading it. For both on-demand viewing and archiving, the digital data containing the audio and video information are stored electronically either in a local digital storage device or through a service such as iTunes U (created to manage, distribute, and control access to educational audio and video content for students within a university).

Although there are a number of important exceptions (see “Intellectual Property Considerations”), webcasting technology is currently used in academia much less than in other areas such as business, law, and medicine. Indeed, webinars are relatively rare for talks given in distinguished seminar series and are almost non-existent for talks presented in more routine seminar series.

**Box 2. Intellectual Property (IP) Permission for Presenters**

Seminar webcasting will involve copyright of the seminar content and copyright of the actual seminar recording. Copyright ownership is governed by several UC copyright policies and depends primarily upon the relationship of the presenter to UC (i.e., Senate or Federation faculty, staff member, student, visitor, or hired presenter). For purposes of this paper, the copyright to the content of most academic seminars will belong to the speaker, while the copyright to that particular recording of the talk belongs to the UC Regents [3].

Streaming live presents few copyright issues so long as the viewing sites are classroom-like, limited to UC viewers, and the speakers sign a consent form allowing the broadcast (see S1). Later broadcast, however, requires more steps. For members of several UC campuses, (Berkeley, Davis and Los Angeles to date) publishing their own presentation can be done relatively easily through iTunes U through a three-step process. Campuses which are not currently participating in iTunes U may have similar tools available. The presenter is responsible for two types of permissions:

*Permission 1*. Individuals who participate in the presentation (i.e., opt in) and could be recognized must voluntarily consent to the recording using a form which is available through the iTunes U process (see Figure S1). It is unnecessary to include individuals who ask occasional short questions or when the videographer pans the room with the camera. UC requires that each unit (department) keep these forms in the event of a later dispute.
*Permission 2*. Any copyright-protected materials that will be audible or visible in the presentation and are owned by someone besides the presenter may require the presenter to obtain a copyright license. If a license is not obtainable, the material may be scrubbed from the recording by campus technology staff prior to distribution.

The recording goes online via a relatively simple process at each campus, which requires certification that permissions 1 and 2 are satisfied. The authority and responsibilities for copyright and IP are allocated differently at each campus, so there can be no common procedure/process for all issues concerned with IP. There is no one unit at most UC campuses (UC Davis is an exception) that addresses all phases of copyright.

Other kinds of direct-to-web exposure in seminars have the potential to create thorny IP problems that are currently unresolved. For example, science-bloggers can—and often do—photograph and immediately post slides on their blog site while attending meetings or seminars. This can create problems for applied researchers who must file patent applications within a year of any information becoming public [4].

**Box 3. Implementing the UC Seminar Network**

Successful development and implementation of the UC Seminar Network concept, much like distance learning [5], depends on simplicity and ease of set up at the venue and lack of any need for special technical expertise [6]. The main operational concepts are buy-in by hosting departments (see example flyer in Figure S2) and prioritizing transmission of content over peripheral concerns such as high-definition images of the speaker, ideal lighting, elaborate speaker introductions, and institutional branding.

The following is a general description of how the proposed UC Seminar Network would work.

**Figure 1:**
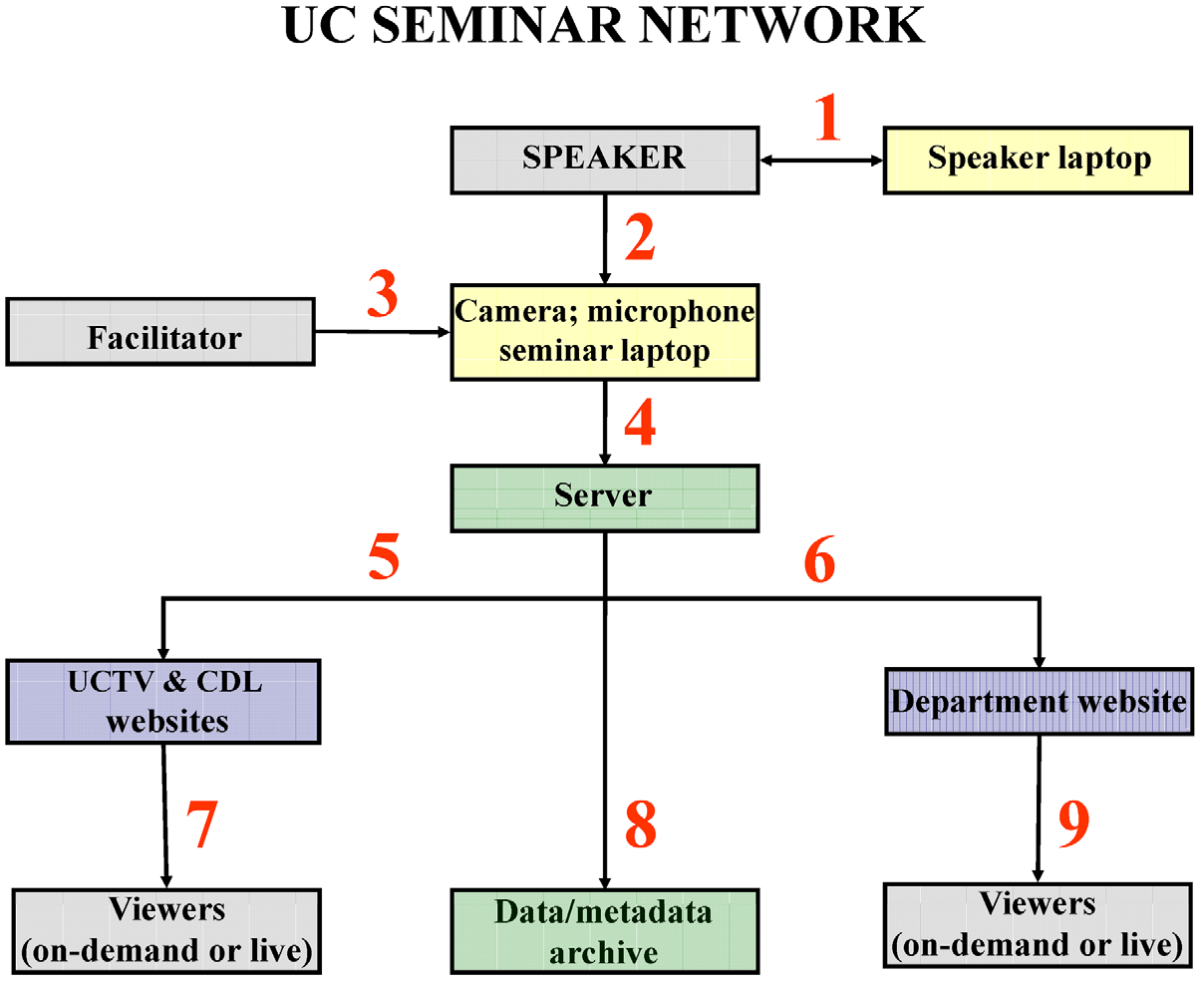
Diagram of infrastructure and data/metadata streams for the proposed UC Seminar Network. Color-coding: gray = person(s); yellow = seminar-related equipment; blue = websites; green = infrastructure software/hardware. Arrows: 1 – PowerPoint slides advanced by speaker through separate (speaker) laptop; 2 – image and voice of speaker captured by camera and microphone coupled to seminar laptop that is linked to server URL; 3 – facilitator (technician or host) in charge of camera and microphone and advancing duplicate copy of PowerPoint slides in synchrony with speaker-controlled seminar laptop; 4 – data captured by camera and microphone and, along with images of speaker's slides, sent to server licensed by the host institution (e.g., Adobe Connect); 5 – metadata (e.g., speaker name, affiliation, and seminar title and abstract) link to data stream (UCTV and CDL refer to University of California Television and the California Digital Library, respectively); 6 – metadata and link to data stream sent to departmental website for live streaming; 7 – live or on-demand viewing through UCTV, CDL, and other websites; 8 – metadata and data stream sorted, cataloged, and archived for later retrieval; limited storage at departmental sites; includes expiration dates for deletion of selected on-demand seminars; primary long-term storage at CDL and UCTV; 9 – live and on-demand viewing through local (departmental) website.

1. Announcements. Key information (host department, speaker/affiliation, seminar title, time, date, location) entered to campus website by sponsoring department or unit along with a URL address for accessing seminar via video streaming. This information would be distributed to a central seminar listing website for all UCs using the Shibboleth Authentication System.
2. Speakers. Seminar speakers will be given an “opt in” choice for having their seminar webcast by requesting that they sign the “consent to record” form. Some committees responsible for distinguished lecturer series offering generous honoraria to speakers might consider mandating webcasting to all invitees.
3. Technology. Minimal requirements for weekly seminar series would involve camera, laptop, microphone, and appropriate video streaming hardware/software. Graduate students (or other designees such as departmental IT person) would set up equipment and log in to the webcasting site. More sophisticated equipment and IT personnel would be used for seminars involving distinguished speakers.
4. Mechanics. Speakers would be required to use the microphone and asked to restrict their movements to the podium area. The tripod-mounted camera operated by a videographer would be trained on the speaker at the podium. The host would be responsible at the start for introducing the speaker and at the end for directing questions from the in-person audience as well as for reading selected emailed queries. Straightforward protocols would be distributed to seminar committees.
5. Video. Beside the option of streaming the video images but not saving them, two levels of recording include: (i) *temporary* for on-demand streaming but with an expiration date e.g., 30, 60, 90 days; and (ii) *archiving* for permanent storage (e.g., in cooperation with the California Digital Library).
6. Capture Data and Infrastructure. The schematic in Figure 1 shows the main categories of activity and equipment in the proposed webcasting system [7] including the flow of digital data from the seminar speaker (presentation) to the video camera and audio equipment (recording/processing) to the server (hosting) and, from there, to the websites for viewer access to live-stream or on-demand viewers (distribution/playback) as well as to the digital archives (storage).

Sponsoring units would be responsible for supplying laptops and video cameras with the campus IT units responsible for software and licensing. Because full-scale implementation (e.g., file hosting, system administration) of the seminar network across all 10 UC campuses will be costly, both internal and external funding sources will need to be identified. However, vetting proof-of-principle and field testing on a limited basis can be done with the existing technology and infrastructure, the results of which will help provide cost estimates and identify constraints.

## Supporting Information

Figure S1 **Generic “consent to record” form to be signed by speakers who opt into UC seminar webcasting.**
(0.03 MB TIF)

Figure S2 **Example of a flyer for promoting departmental participation in seminar webcasting at the University of California.**
(3.18 MB TIF)

## References

[1] Evans J. A, Reimer J (2009). Open access and global participation in science.. Science.

[2] Ha L, Ganahi R. J (2007). Webcasting worldwide: business models of an emerging global medium.

[3] Additional details of the UC intellectual property and copyright compliance policies are available at http://webcast.berkeley.edu/wp/policies/#copyright

[4] Brumfiel A (2009). Breaking the convention?. Nature.

[5] Smith M. S (2009). Opening education.. Science.

[6] Westwood M. A, Flett A. S, Riding P, Moon J. C (2009). How to webcast lectures and conferences.. Br Med J.

[7] Deal A (2007). Lecture webcasting. Teaching with technology white paper series.. http://www.cmu.edu/teaching/resources/PublicationsArchives/StudiesWhitepapers/LectureWebcasting_Jan07.pdf.

