# Supplementary figures and images for: University of California Research Seminar Network: A Prospectus
Source: PLoS Biol. 2010 Jan 19;8(1):e1000289. doi: 10.1371/journal.pbio.1000289 (PMC2799637; doi:10.1371/journal.pbio.1000289)

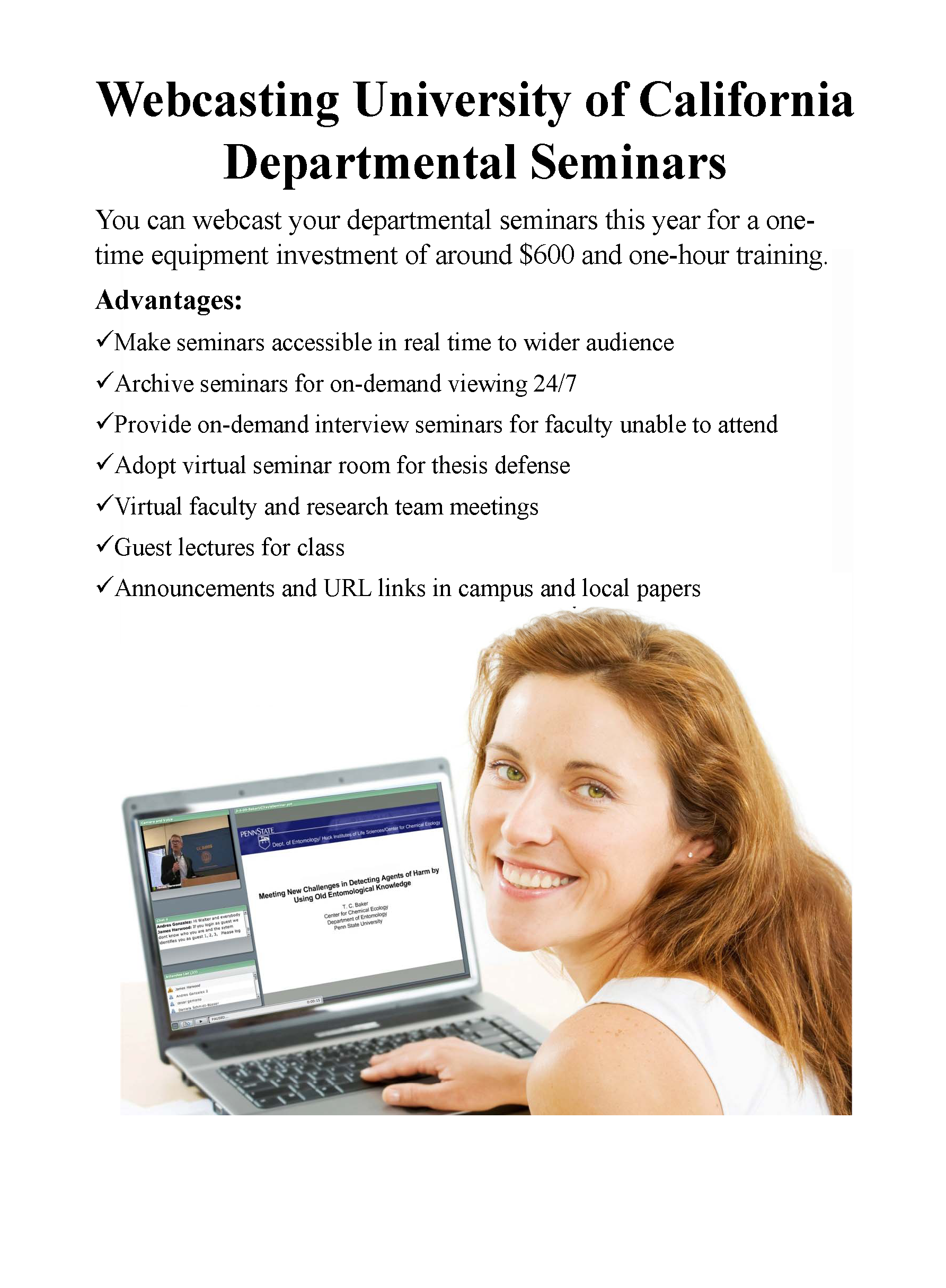

Supplement: Figure S2 — Example of a flyer for promoting departmental participation in seminar webcasting at the University of California. (3.18 MB TIF) [file pbio.1000289.s002.tif]
